# Predicting refractoriness in lateral epicondylitis using initial grip strength and quickdash: a retrospective cohort study

**DOI:** 10.1186/s12891-025-08902-7

**Published:** 2025-07-04

**Authors:** Kazuhiro Ikeda, Akira Ikumi, Shinzo Onishi, Takeshi Ogawa, Sho Kohyama, Yuichi Yoshii

**Affiliations:** 1 Institute of Clinical Medicine, Department of Orthopedic Surgery, University of Tsukuba, 1-1 Tennodai, Ibaraki Prefecture, 305-8577 Japan; 2 Department of Orthopedic Surgery, Kikkoman General Hospital, Chiba Prefecture, Japan; 3 Department of Orthopedic Surgery, NHO, Mito Medical Center, Ibaraki Prefecture, Japan; 4 Department of Orthopedic Surgery, Tokyo Medical University Ibaraki Medical Center, Ibaraki Prefecture, Japan

**Keywords:** Lateral epicondylitis, Refractory, Chronic, Grip strength

## Abstract

**Background:**

Lateral epicondylitis is a self-limited disease and refractory condition; thus providing optimal treatment is challenging. This study investigated a method for predicting cases that would not improve sufficiently under a wait-and-see policy using clinical indicators obtained during the initial consultation.

**Methods:**

Twenty-two patients with lateral epicondylitis prescribed a resting orthosis and followed up for 6 months were included. Grip strength ratios for affected/unaffected side; quick disabilities of the arm, shoulder, and hand (QuickDASH) scores were measured at 6-week intervals. Receiver operating characteristic curves for predicting refractory cases were created from the initial measurement items to determine the cut-off values and prediction accuracy.

**Results:**

The 6-month post-treatment QuickDASH scores for the 14 improved patients and 8 refractory patients were 2.3 ± 4.7 and 25.9 ± 15.4, respectively. Grip strength ratios significantly predicted refractoriness risk with a 0.54 cut-off value. The QuickDASH scores significantly predicted refractoriness risk with a 30-point cut-off value. Meeting either of these cutoff values achieved a sensitivity of 1.0 for predicting refractoriness.

**Conclusions:**

The patients with a grip strength ratio ≤ 0.5 on the affected side or a QuickDASH score ≥ 30 at initial consultation continued having symptoms 6 months after conservative treatment.

**Supplementary Information:**

The online version contains supplementary material available at 10.1186/s12891-025-08902-7.

## Background

Lateral epicondylitis (LE) is a tendinopathy of the forearm extensor muscles [1, 2]. It is common among laborers performing gripping tasks involving forearm pronation, with prevalence rates of 2–5% [1, 3, 4]. Patients with LE experience decreased grip strength due to lateral elbow pain [5]. Approximately 80–90% of patients with LE show improvement within 6–12 months [1, 6–8]. For patients who can obtain the required rest by avoiding pain-inducing activities, a “wait and see” policy is the basic approach in several countries [8]; therefore, LE is often referred to as a self-limiting disease [7]. However, approximately 10–20% of patients present with prolonged symptoms and those who do not improve within 6 months are classified as having refractory LE [1, 6]. Various factors including sex, smoking, labor, psychiatric disorders, pain sensitivity, local steroid injection, and histological severity contribute to refractoriness [3, 6, 8–12]. Refractory LE significantly impairs patients’ ability to work over extended periods, imposing a substantial economic burden on individuals, corporations, and the national economy [13]. Preventing LE refractoriness is a societal challenge that requires appropriate interventions based on systematic treatment tailored to the disease severity and risk of refractory status.

However, diverse LE treatment options, including non-intervention observation, orthotic therapy, steroid injections, percutaneous needling, and extracorporeal shock wave therapy, have not been systematically adopted. Since the reported risk factors of refractory LE are primarily qualitative assessments [3, 6, 8–12], it is difficult to quantify an individual’s refractory risk. Accordingly, treatment decisions are often made subjectively by the clinicians [1, 6, 7, 9], which may lead to suboptimal outcomes and inefficient use of medical resources due to under- or over-treatment. Therefore, quantitative indicators for refractoriness are essential to improve treatment outcomes and efficiently utilize medical resources.

Given these challenges, we explored whether clinical severity at initial consultation could serve as a practical indicator for predicting refractoriness because it reflects a range of patient-related factors. Working patients without sufficient social support are expected to have poor treatment compliance, which may lead to delayed medical consultation and worsening of LE symptoms [12]. Prolonged disease duration may exacerbate histological degeneration at the wrist extensor origin, resulting in higher clinical severity and greater challenges in disease control [2, 6, 14, 15]. Conversely, patients with severe symptoms, despite not having life circumstances that exacerbate their condition, may exhibit heightened pain sensitivity and poorer outcomes [5, 10, 11, 16, 17]. Overall, initial clinical severity may reflect multiple underlying factors that influence treatment responsiveness and long-term prognosis for LE.

This study was designed as an exploratory investigation to identify practical clinical indicators that may help predict individual risk of refractoriness. The objective was to generate hypotheses regarding potentially useful prognostic indicators that could inform future, larger-scale validation studies.

## Methods

### Study design and participants

This was a retrospective cohort study (Level III evidence). Clinical data were prospectively collected by a single clinician (KI) at a secondary care center and subsequently analyzed retrospectively to assess the prognostic utility of initial clinical severity in patients undergoing conservative treatment for LE.

The study protocol conforms to the principles outlined in the 1964 Declaration of Helsinki. Our Institutional Review Board approved this study (Approval No. KC-H26; Date: February 5, 2022), and we obtained written informed consent from all the participants.

The study included patients diagnosed with unilateral LE who initiated conservative treatment between February 2022 and July 2023. The exclusion criteria were upper-limb disorders other than LE and a history of fractures or dislocations, osteoarthritis, rheumatoid arthritis, or psychiatric disorders. During the study period, 35 patients (37 elbows) were diagnosed with LE. Among them, 13 patients were excluded: two patients with bilateral involvement, one patient with polymyalgia rheumatica, one patient with concomitant medial epicondylitis, three patients who refused participation, and six patients who discontinued treatment within the 6-month study period. Details about these dropout cases are provided in Supplemental Table S1.

Consequently, this study comprised 22 patients with unilateral LE (9 men and 13 women; average age 58.0 ± 12.5 years, height, 161.8 ± 8.0 cm; weight, 63.8 ± 14.4 kg; and body mass index, 24.3 ± 4.4).

### LE diagnosis and treatment

All diagnoses of LE were determined by a single clinician (KI, a board-certified hand surgeon certified by the Japanese Society for Surgery of the Hand) using standardized diagnostic criteria, which included tenderness at the lateral epicondyle of the humerus and positive findings for at least two of the following assessments: the Thomsen test, Maudsley’s test, and lateral elbow pain during gripping [18, 19]. Elbow radiography was performed for all patients to rule out trauma and arthritic conditions.

**Fig. 1:**
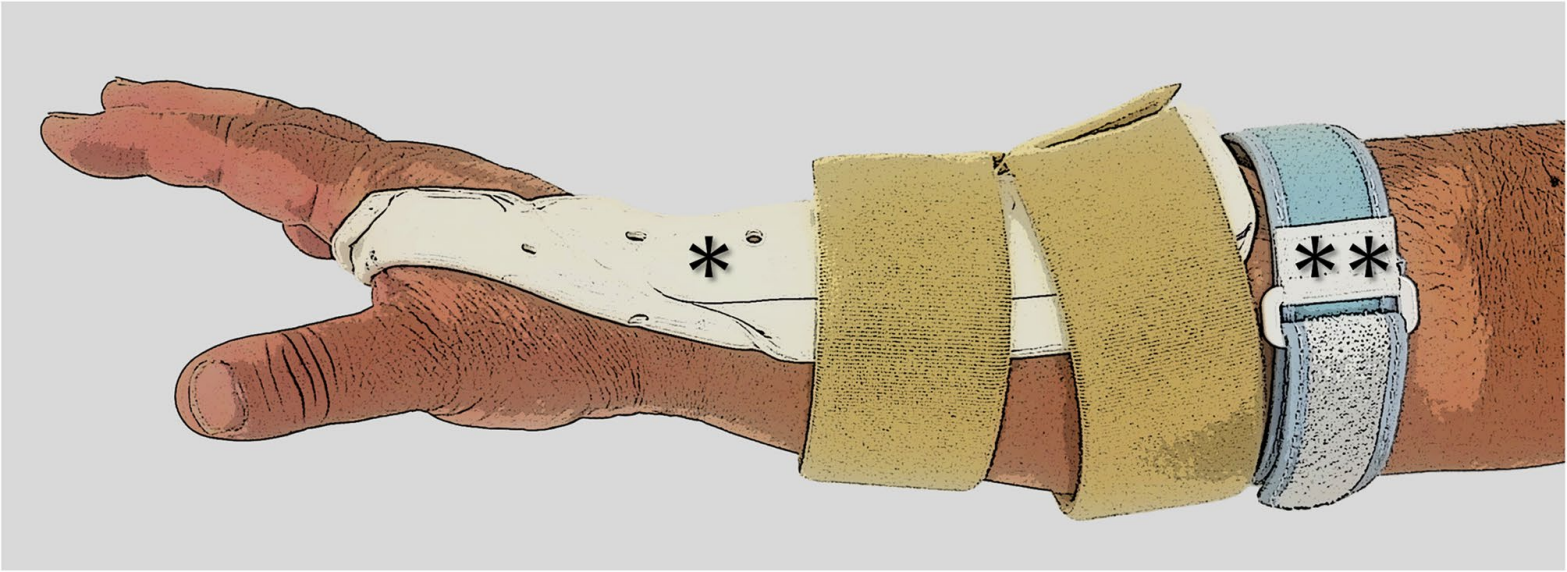
Orthoses This image demonstrates how a dorsal cock-up splint (*) combined with a tennis elbow brace (**) is worn. The tennis elbow brace is positioned at the maximum circumference of the forearm and tightened with the maximum strength that avoids skin pinching and discomfort, as instructed

Although orthotic devices were prescribed to promote rest, the fundamental treatment approach was to implement a wait-and-see policy. All patients were prescribed a tennis elbow brace (tennis elbow supporter; ALCARE Co., Ltd., Tokyo, Japan) and dorsal 30-degree cock-up splint (created by an occupational therapist) at the initial consultation. Patients were instructed to wear the orthoses as much as possible during daily activities, except while sleeping, performing stretching exercises, or in situations where the hand could get wet, such as bathing. Instructions on correct positioning and fitting of the orthoses were provided by an occupational therapist during the initial consultation (Fig. 1). The treating physician conducted follow-up appointments with the patients at 6-week intervals until they no longer met the diagnostic criteria for LE. In coordination with the doctor appointment, the occupational therapist provided daily living guidance as well as instructions regarding stretching and massage to relax the forearm extensor muscles. Analgesic and local steroid injections to forearm extensors origins were not included in the treatment protocol.

### Evaluation items

At the initial consultation, baseline characteristics were recorded including known risk factors for refractoriness (sex, manual labor) [3, 6, 8–12], as well as age, height, weight, BMI, illness duration, and dominance of the affected limb. Illness duration was defined as the period from self-reported onset of lateral elbow pain to the initial consultation. Patients were asked to report the approximate time of symptom onset, and the duration was recorded in weeks to account for potential recall bias.

As for LE clinical severity measures that can be easily evaluated in clinical settings, this study utilized grip strength; the Quick Disabilities of the Arm, Shoulder, and Hand (QuickDASH) scores. Grip strength is used as an objective indicator for wrist dorsiflexion weakness and pain [1, 5, 20]. Grip strength was measured once during each examination to minimize patient discomfort and to avoid exacerbating pain, which could have influenced the measurement results for patients with LE. It was assessed using a hand dynamometer (T.K.K.5001^®^; Takei Scientific Instruments Co., Ltd., Niigata, Japan) with the upper limb hanging down, the elbow extended, and the forearm in a neutral position, which is a standardized method commonly used in clinical and research settings for assessing grip strength [5, 11]. The patients were instructed to grip the dynamometer with the maximum force achievable despite the pain [6]. Grip strength was measured once per examination to reduce pain-related bias, and the grip strength ratio for the affected/unaffected sides was subsequently calculated [5, 11]. Additionally, visual analogue scale (VAS) scores during activities of daily living (ADL) were collected as a supplemental measure to assess patients’ subjective pain levels (Supplemental Table S2). Patients completed the QuickDASH and VAS questionnaires outside the examination room to avoid potential influence from the clinician’s presence.

When predicting refractory cases in an initial consultation, it is desirable to increase sensitivity rather than specificity to avoid missing refractory cases. Accordingly, we defined refractory cases as those meeting the aforementioned diagnostic criteria for LE at 6 months after treatment. Cases in which the diagnostic criteria for LE became negative within 6 months of treatment initiation were defined as improved cases. Patients who showed early improvement were observed until 6 months if they consented. Patients who did not show improvement and discontinued treatment within 6 months were excluded from the study.

### Statistical analyses

Due to the small sample size and concern for low statistical power, normality testing was omitted. Data are expressed as median (interquartile range [IQR]). Missing values in time-series data were imputed using linear interpolation when both preceding and subsequent data points were available; otherwise, the group median was substituted for items without adjacent data.

Categorical variables were compared between the groups using the chi-square test, and continuous or ordinal data were compared using the Mann–Whitney U test. Two-tailed tests were used for comparisons of baseline patient characteristics, whereas one-tailed tests were applied to severity indicators (grip strength ratio, QuickDASH score) to assess whether the refractory group had worse outcomes.

Receiver operating characteristic (ROC) curves were created for predicting refractoriness based on the grip strength ratios and QuickDASH scores at the initial consultations. The cut-off values on the ROC curve were determined as the points closest to the top-left corner. Subsequently, we evaluated the sensitivity and specificity of a combined criterion, defined by meeting either the cut-off value for the grip strength ratio or the QuickDASH score. Correlation between the grip strength ratio and QuickDASH scores were assessed using Spearman’s correlation coefficient. Correlation coefficients of ± 0.3 < *r* < ± 0.7 and r ≥ ± 0.7 were considered to indicate moderate and strong correlations, respectively. Statistical significance was set at *p* < 0.05.

A post hoc sample size analysis was performed to assess the adequacy of the enrolled sample size based on the actual predictive performance (AUC) observed in the study. The statistical analyses were performed using EZR ver. 1.65 (Saitama Medical Center, Jichi Medical University, Saitama, Japan).

## Results

### Demographic and clinical characteristics

The improved group included 14 patients, median age 59 (54–71) years; 8 men, 6 women; median QuickDASH score at 6 months post-treatment was 1.1 (0–4.0). The refractory group included 8 patients: median age 47 (44–54) years; 3 men, 5 women; QuickDASH score at 6 months post-treatment was 21.6 (14.8–31.8). Patients in the refractory group were significantly younger than those in the improved group (*p* = 0.027); however, age did not contribute to the risk of refractoriness in the univariate analysis (*p* = 0.053). No significant differences were observed between groups for other background characteristics, including known risk factors for refractoriness (Table 1).

**Table 1:** Patient demographic and clinical characteristics

Group	Improved (*n* = 14)	Refractory (*n* = 8)	*p*-value
Age (years)	59 (54–71)	47 (44–54)	*0.014*
Sex^a^	men: 6 women: 8	men: 3 women: 5	0.806
Height (cm)	160 (156–164)	163 (159–169)	0.346
Weight (kg)	60 (56–63)	65 (51–76)	0.083
Body mass index	22.8 (21.2–25.9)	25.0 (20.3–27.8)	0.942
Illness duration (weeks)	7 (3–16)	10 (3–13)	0.911
Affection in the dominant hand (%)	78.6	75.0	0.848
Engaging in physical labor (%)^a^	50	87.5	0.079

Data are presented as median (interquartile range)

^a^Known risk factors

### Clinical course

**Fig. 2:**
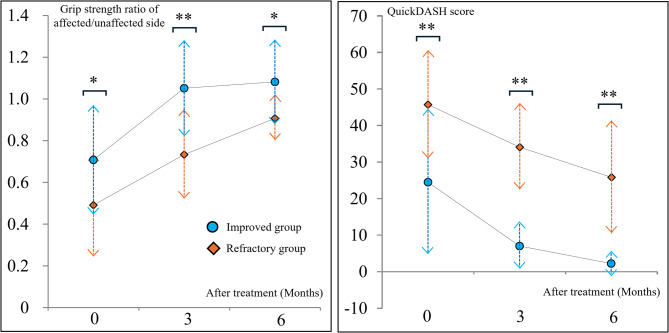
Clinical course for each group The evaluation items were compared between the improved and refractory groups at each follow-up month QuickDASH, Quick Disabilities of the Arm, Shoulder, and Hand. *, *p* < 0.05; **, *p* < 0.01

Figure 2; Table 2 present details regarding the clinical courses of the improved and refractory groups. The grip strength ratio was significantly lower in the refractory group than those in the improved group throughout the observation period. Similarly, the QuickDASH scores were significantly higher in the refractory group than those in the improved group throughout the observation period.

**Table 2:** Detailed parameters regarding the clinical course

	Group	0 months	3 months	6 months
Grip strength ratio^a^	Improved	0.68 (0.58–0.90)	1.01 (0.92–1.15)	1.00 (0.98–1.12)
	Refractory	0.47 (0.36–0.56)	0.67 (0.59–0.92)	0.92 (0.83–1.00)
	*p-*value	*0.033*	*0.003*	*0.014*
QuickDASH score	Improved	18.7 (12.0–35.0)	8.3 (0.6–10.8)	1.1 (0–4.0)
	Refractory	47.8 (34.3–51.7)	34.1(25.0–38.6)	21.6 (14.8–31.8)
	*p-*value	*0.005*	*< 0.001*	*< 0.001*

Data are presented as median (interquartile range)

^a^Grip strength ratio = grip strength (affected/unaffected side)

*QuickDASH *Quick Disabilities of the Arm, Shoulder, and Hand

### Course of patients with refractory LE

The course of the patients with refractory LE after 6 months of treatment was as follows: Two patients improved over time with continued use of the same orthotic treatment; one became symptom free at 8 months and the other at 15 months post-treatment. Two patients with persistent symptoms switched to a wrist dorsiflexion functional orthosis, and both of them became symptom free after 12 months. One patient with severe symptoms and significant magnetic resonance imaging (MRI) signal changes of common extensor tendon origin underwent Nirschl surgery at 6 months post-treatment, becoming symptom free by 9 months post-surgery. Two patients discontinued treatment before confirming symptom resolution. One patient discontinued visits due to lung cancer treatment.

### ROC curves and correlations

**Fig. 3:**
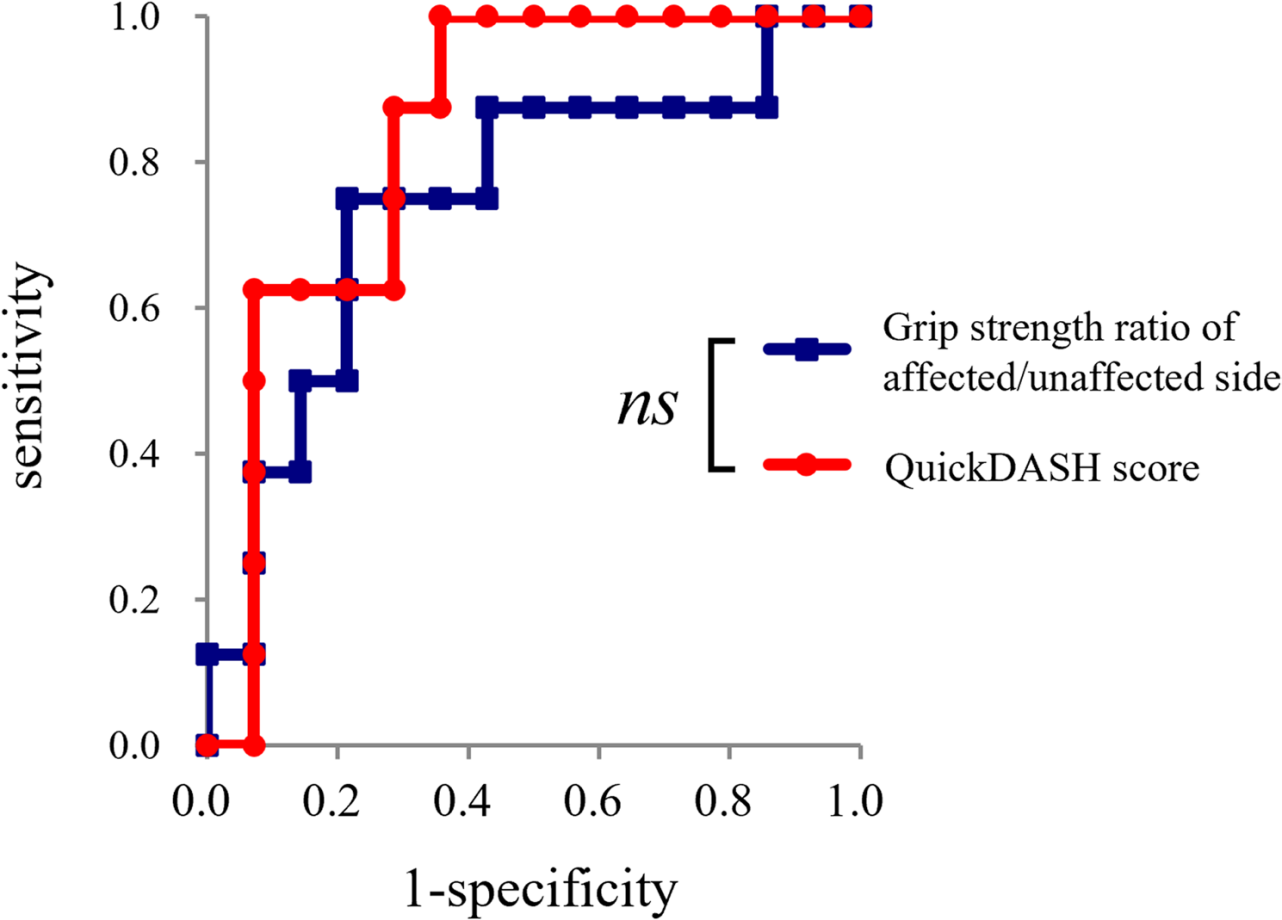
ROC curve predicting refractoriness based on clinical severity at the initial visit These ROC curves used the grip strength ratio of the affected to unaffected side and the QuickDASH score as explanatory variables. Their detailed parameters are presented in Table 3 ns, not significant; QuickDASH, Quick Disabilities of the Arm, Shoulder, and Hand; ROC, receiver operating characteristic

The ROC curves for predicting refractoriness using the grip strength ratio and QuickDASH scores at the initial consultations are shown in Fig. 3; further details are presented in Table 3. The grip strength ratio significantly predicted the risk of refractoriness, with a cut-off value of 0.54. Additionally, the QuickDASH score significantly predicted the risk of refractoriness, with a cut-off value of 30 points. There was no significant difference in the AUC of the ROC curves between the grip strength ratio and QuickDASH score (*p* = 0.530). When a combined criterion of either a grip strength ratio ≤ 0.54 or a QuickDASH score ≥ 30 was applied, the sensitivity and specificity for predicting refractoriness were 1.00 and 0.57, respectively.

**Table 3:** Details regarding the ROC curve

	AUC		Cutoff					
	AUC (95% CI)	*p*-value	Cutoff	Sens	Spec	OR	PLR	NLR
Grip strength ratio^a^ (a)	0.75 (0.52–0.98)	*0.035*	0.54	0.75	0.79	11.0	3.50	0.32
QuickDASH score (b)	0.84 (0.67–1.01)	*< 0.001*	30	0.88	0.79	17.5	3.06	0.18
(a) ≤ 0.54 or (b) ≥ 30				1.00	0.57	-	2.33	0

^a^Grip strength ratio = grip strength (affected/unaffected side)

*AUC *Area under the ROC curve, *95% CI *95% confidence interval, *NLR *Negative likelihood ratio, *OR *Odds ratio, *PLR *Positive likelihood ratio, *QuickDASH *Quick disabilities of the arm, shoulder, and hand, *ROC *Receiver operating characteristic, *Sens *Sensitivity, *Spec *Specificity

The correlation coefficient between the grip strength ratio and QuickDASH score was − 0.62, indicating a moderate negative correlation.

Based on the observed AUC values of 0.75 for the grip strength ratio and 0.84 for the QuickDASH score, a post hoc sample size analysis was performed. Assuming an AUC of 0.75–0.84, with a type I error of 5% and a power of 80%, a sample size of 8–15 cases per group was determined to be sufficient.

## Discussion

This study demonstrated that the risk of refractory LE could be predicted based on the initial grip strength and QuickDASH score. Patients whose initial grip strength was less than half of that in the unaffected side and those with QuickDASH scores ≥ 30 were at risk of refractory LE. Smidt et al. [8] reported a remission rate of 76% after 6 months and 78% after 1 year with a “wait and see” policy, which indicates that patients showing no improvement by 6 months are unlikely to experience natural remission. For those with poor compliance, social support is essential to improve the treatment environment and reduce patient financial burdens. This support includes issuing medical certificates to recommend work leave or modifications and preparing reports for worker compensation to secure wage and treatment cost coverages. Collaboration with occupational physicians is crucial to address workplace challenges and support treatment adherence. For patients with significant histological degeneration at the attachment sites of the forearm extensors, it is important to consider treatments that promote histological repair such as surgery, extracorporeal shock wave therapy, and platelet-rich plasma injections. Quantitative MRI evaluation methods can predict histological degeneration severity [6]. Future studies are warranted to investigate the effectiveness of initial active interventions for high-risk patients.

A notable aspect of this study was the practicality of the selected clinical indicators. Grip strength testing is commonly performed in orthopedic practice and allows for simple and quick evaluation. Similarly, the QuickDASH score is a widely used patient-reported outcome measure that can be completed by patients while waiting for their consultation. Accordingly, grip strength and the QuickDASH score are extremely practical indicators of refractory risk that can be assessed even in busy clinical settings. Furthermore, combining these objective and subjective indicators (grip strength ratio ≤ 0.54 or QuickDASH score ≥ 30) enabled us to achieve a sensitivity of 1.00 for predicting refractoriness. This result suggests that a straightforward screening strategy based on these two indicators could effectively identify high-risk patients and offer opportunities for early additional evaluation, closer monitoring, and proactive intervention.

The complementary nature of these two measures may further enhance their clinical utility. The moderate correlation between grip strength and the QuickDASH score suggests that reduced grip strength and pain during gripping are directly related to ADL limitations. Since the grip strength ratio cannot be assessed in patients with bilateral involvement, it is important to evaluate the refractory risk from multiple perspectives.

A limitation of this study is that a multivariate analysis was not performed; thus potential confounding factors could not be rigorously addressed. Due to the limited sample size, this exploratory study was limited to direct comparisons of known risk factors between the improved and refractory groups, without adjusting for potential confounders. The small sample size may also have affected the robustness and generalizability of the findings. Although statistically significant AUC values suggested a reduced risk of β-error, further validation with larger, adequately powered studies is required to confirm these results. Future large-scale prospective studies are needed to externally validate these findings and confirm their generalizability. Additionally, grip strength can be influenced by factors such as handedness, occupation, and sports history [21–23], which should be considered when interpreting the grip strength ratio.

## Conclusions

Both the grip strength ratio and the QuickDASH score at the initial consultation demonstrated potential utility for predicting refractory LE. Using both an objective indicator (grip strength) and a subjective indicator (QuickDASH) may help minimize the risk of missing high-risk cases during early screening. Patients whose grip strength has declined to half of that for the healthy side, or who have a QuickDASH score ≥ 30 at the initial consultation, should be carefully monitored and managed with consideration for refractoriness.

## Supplementary Information

Supplementary Material 1.

Supplementary Material 2.

## Data Availability

The datasets used and/or analyzed in the present study are available from the corresponding author upon reasonable request.

## Abbreviations

ADL: Activities of daily living
AUC: Area under the ROC curve
LE: Lateral epicondylitis
MRI: Magnetic resonance imaging
QuickDASH: Quick disabilities of the arm, shoulder, and hand
ROC: Receiver operating characteristic
VAS: visual analogue scale

## Declarations

Written informed consent to participate in this study was obtained from all the patients.
