# Supplementary material for: Predicting refractoriness in lateral epicondylitis using initial grip strength and quickdash: a retrospective cohort study
Source: BMC Musculoskelet Disord. 2025 Jul 4;26:645. doi: 10.1186/s12891-025-08902-7 (PMC12228226; doi:10.1186/s12891-025-08902-7)
Supplement: Supplementary file 1 — Supplementary Material 1. [file 12891_2025_8902_MOESM1_ESM.docx]

**Supplemental Table 1 Dropout Cases**

| Cases | Age  (years) | Sex | Occupation | Final Observation (duration from initial consultation) | Severity (initial/final observation) | | |
| --- | --- | --- | --- | --- | --- | --- | --- |
|  |  |  |  |  | Grip strength ratio of affected/ unaffected side | QuickDASH score | VAS |
| 1 | 43 | Man | Clerk | 6 weeks | 0.63/ 0.87 | 20.5/18.2 | 27/ 15 |
| 2 | 67 | Woman | Driver | 0 weeks | 0.22/ NA | 55/ NA | 50/ NA |
| 3 | 47 | Man | Sales | 0 weeks | 0.72/ NA | NA*/ NA | 70/ NA |
| 4 | 50 | Man | Manufacturing industry | 6 weeks | 0.77/ 1.00 | 66.1/12.5 | 40/ 10 |
| 5 | 40 | Man | Manufacturing industry | 6 weeks | 1.17/ 0.89 | 15/11.4 | 70/ 5 |
| 6 | 44 | Woman | Waitress | 6 weeks | 0.63/ 0.29 | 34.1/NA* | 60/ 75 |

*, not submitted; NA, not applicable; QuickDASH, quick disabilities of the arm, shoulder, and hand; VAS, visual analogue scale.
