# Supplementary material for: Predicting refractoriness in lateral epicondylitis using initial grip strength and quickdash: a retrospective cohort study
Source: BMC Musculoskelet Disord. 2025 Jul 4;26:645. doi: 10.1186/s12891-025-08902-7 (PMC12228226; doi:10.1186/s12891-025-08902-7)
Supplement: Supplementary file 2 — Supplementary Material 2. [file 12891_2025_8902_MOESM2_ESM.docx]

**Supplemental Table S2 VAS scores**

| Group | 0 months | 3 months | 6 months |
| --- | --- | --- | --- |
| Improved | 50.0 (41.3–74.3) | 6 (0–24.8) | 0 (0–1.5) |
| Refractory | 57.0 (50.0–66.3) | 41.5 (32.5–53.3) | 29.5 (17.8–47.3) |
| *p-*value | 0.392 | 0.002 | < 0.001 |

VAS, visual analogue scale.
